# Integrating Genome-Wide Association Studies With Pathway Analysis and Gene Expression Analysis Highlights Novel Osteoarthritis Risk Pathways and Genes

**DOI:** 10.3389/fgene.2019.00827

**Published:** 2019-09-13

**Authors:** Feng Gao, Yu Yao, Yiwei Zhang, Jun Tian

**Affiliations:** Department of Trauma and Emergency Surgeon, The Second Affiliated Hospital, Harbin Medical University, Harbin, China

**Keywords:** osteoarthritis, genome-wide association study, pathway analysis, expession quantitative trait loci analysis, gene expression analysis

## Abstract

Osteoarthritis (OA) is the most common degenerative joint disorder worldwide. To identify more genetic signals, genome-wide association study (GWAS) has been widely used and elucidated some OA susceptibility genes. However, these susceptibility genes could only explain only a small part of heritability of OA. It is suggested that the identification of disease-related pathways may contribute to understand the genomic etiology of OA. Here, we integrated the GWAS into pathway analysis to identify novel OA risk pathways. In this study, we first selected 187 independent genetic variants identified by GWAS (*P* < 1.00E−05) and found that most of these genetic variants are noncoding mutations. We then conducted an expression quantitative trait loci analysis and found that 165 of these 187 genetic variants could significantly regulate the expression of nearby genes. Third, we identified OA susceptibility genes corresponding to these genetic variants, conducted a pathway analysis, and identified novel OA-related KEGG pathways, GO biological processes, GO molecular functions, and GO cellular components. In KEGG database, transforming growth factor β signaling pathway is the most significant signal (*P* = 5.98E−05) and is the only pathway after the BH multiple-test adjustment with false discovery rate (FDR) = 0.02. In GO database, we identified 24 statistically significant GO biological processes, one statistically significant GO molecular function, and five statistically significant GO cellular components (FDR < 0.05). These signals are related with chondrocyte differentiation and development, which are all known biological pathways associated with OA. Finally, we conducted an OA case–control gene expression analysis to evaluate the differential expression of these OA risk genes. Using an OA case–control gene expression analysis, we showed that 44 risk genes were suggestively differentially expressed in OA cases compared with controls (*P* < 0.05). Three genes, WWP2, COG5, and MAPT, were statistically differentially expressed in OA cases compared with controls (*P* < 0.05/122 = 4.10E−04). Hence, our findings may contribute to understanding the genomic etiology of OA.

## Introduction

Osteoarthritis (OA) is the most common degenerative joint disorder worldwide (Martel-Pelletier et al., 2016). Osteoarthritis could cause pain and disability in elderly people (Parmet et al., 2003; Zeggini et al., 2012). It is considered that OA is caused by the combination of risk factors with increasing age and obesity (Parmet et al., 2003; Martel-Pelletier et al., 2016). Osteoarthritis is a complex disease and has a strong genetic component (Zeggini et al., 2012). To identify more genetic signals, genome-wide association study (GWAS) has been widely used and elucidated some OA susceptibility genes (Zeggini et al., 2012; Styrkarsdottir et al., 2014; Styrkarsdottir et al., 2018; Zengini et al., 2018; Tachmazidou et al., 2019). However, these susceptibility genes could explain only a small part of heritability of OA (Zeggini et al., 2012; Styrkarsdottir et al., 2014; Styrkarsdottir et al., 2018; Zengini et al., 2018; Tachmazidou et al., 2019). It is suggested that the identification of disease-related pathways may contribute to understand the genomic etiology of OA (Cibrian Uhalte et al., 2017).

In recent years, kinds of bioinformatics software and databases have been developed, especially for the identification of disease-related pathways such as VEGAS (Versatile Gene-based Association Study) (Liu et al., 2010), INRICH (INterval enRICHment analysis) (Lee et al., 2012a), and GSA-SNP2 (Yoon et al., 2018). Meanwhile, these methods have been widely used in OA-related disease rheumatoid arthritis (RA) (Eleftherohorinou et al., 2009; Eleftherohorinou et al., 2011; Lee et al., 2012b; Song et al., 2013; Zhang et al., 2016), but not in OA. Here, we first selected all significant OA risk variants identified by GWAS and evaluated their distribution in genome coding and noncoding regions. Second, we then conducted an expression quantitative trait loci (eQTLs) analysis to evaluate the effect of these genetic variants on gene expression. Third, we performed a pathway analysis of OA susceptibility genes detected by OA-related genetic pathways. Finally, we conducted an OA case–control gene expression analysis to evaluate the differential expression of these OA risk genes.

## Materials and Methods

### Selection of OA Risk Variants

We selected the potential OA risk variants identified by GWAS by searching for the NHGRI GWAS catalog database using the keyword “osteoarthritis” (Welter et al., 2014; MacArthur et al., 2017; Buniello et al., 2019). Finally, we selected 187 genetic variants associated with OA (*P* < 1.00E−05) (Welter et al., 2014; MacArthur et al., 2017; Buniello et al., 2019). These variants are associated with OA, OA of the hip, OA of the knee, or OA of the hand. Here, we provided all related information about these variants in **Supplementary Table 1**.

### Identification of OA Risk Genes

For each of these 187 genetic variants, if this variant is an intronic variant, we select its corresponding gene. If this variant is an intergenic variant, we select its corresponding nearest upstream and downstream genes. The gene set consists of 202 risk genes using all these 187 genetic variants, as provided in **Supplementary Table 1**.

### Function Analysis

We used the HaploReg tool (v4.1) to annotate these 187 genetic variants and evaluate how many genetic variants are coding and noncoding mutations (Ward and Kellis, 2012; Ward and Kellis, 2016). The reference information is from the 1000 Genomes Project (Ward and Kellis, 2012; Ward and Kellis, 2016).

### eQTLs Analysis

When noncoding genetic variants are identified, we conducted an eQTLs analysis to evaluate the effect of these genetic variants on gene expression using Phenol Scanner (v2), a database of human genotype–phenotype associations (Staley et al., 2016; Kamat et al., 2019).

### Identification of OA Risk Pathways

Here, we selected the pathway resources from KEGG and GO databases, as provided in Web-based Gene Set Analysis Toolkit (WebGestalt version 2019) (Wang et al., 2017). To identify a potential OA risk pathway, the hypergeometric test was used to evaluate an overrepresentation of the OA risk genes among all the genes in the selected pathway, as did in previous studies (Bao et al., 2015; Zhao et al., 2015; Jiang et al., 2017; Liu et al., 2017; Wang et al., 2017). For a given pathway, the *P* value of observing more than *K* OA risk genes could be calculated by

$$P=1-\sum_{i=0}^{K}\frac{\left(\begin{array}{c} S\\ i \end{array})(\begin{array}{c} N-S\\ m-i \end{array}\right)}{\left(\begin{array}{c} N\\ m \end{array}\right)}ß$$

where *N* is the total number of genes that are of interest (the reference gene list), *S* is the number of all OA risk genes (here, n = 202), *m* is the number of genes in the pathway, and *K* is the number of OA risk genes in the pathway, as did in previous studies (Liu et al., 2012; Liu et al., 2013; Liu et al., 2014; Quan et al., 2015; Shang et al., 2015; Xiang et al., 2015).

Using WebGestalt version 2019, the minimum number of genes for a category is 5, and the maximum number of genes for a category is 2000. In addition, we used the BH multiple-test adjustment method to adjust the *P* value of each pathway. A specific pathway with adjusted FDR < 0.05 is considered to be statistically significant. A specific pathway with unadjusted *P* < 0.05 is considered to be suggestively significant.

### OA Case–Control Gene Expression Analysis

We conducted an OA case–control gene expression analysis to evaluate the differential expression of these 202 risk genes, as provided in **Supplementary Table 1**. The gene expression profile dataset is from peripheral blood mononuclear cells of 106 OA patients and 33 sex and age-matched healthy controls, which is a subset of the Genetics osteoarthritis and Progression study (Ramos et al., 2014). Here, we used the interactive web tool GEO2R to identify genes that are differentially expressed in OA patients and healthy controls. A gene with unadjusted *P* < 0.05 is considered to be suggestively differentially expressed. In addition, we used the Bonferroni correction method to adjust these *P* values and define the statistically differential expression, as described in a previous study (Krzywinski and Altman, 2014).

## Results

### Function Analysis

Function analysis using HaploReg tool (v4.1) showed that eight genetic variants, rs12193102, rs35611929, rs375575359, rs528981060, rs532464664, rs541612392, rs547116051, and rs547181612, were not found in 1000 Genomes Phase 1 data. In the remaining 179 genetic variants, 11 genetic variants are coding mutations, and the other 168 genetic variants are noncoding mutations. More detailed information is provided in **Supplementary Table 2**.

### eQTLs Analysis

Using Phenol Scanner (v2), the eQTLs analysis showed that 165 of these 187 genetic variants could significantly regulate the expression of nearby genes with *P* < 0.01. We found that the other 22 genetic variants were not eQTLs including rs111427307, rs11335718, rs11409738, rs12028630, rs12193102, rs138063419, rs143083812, rs150198051, rs201708019, rs35087650, rs35912128, rs375575359, rs4867568, rs528981060, rs541612392, rs547116051, rs547181612, rs5834501, rs62262139, rs6557013, rs7510312, and rs9930333. More detailed information is provided in **Supplementary Table 3**.

### Identification of OA-Related KEGG Pathways

Using these 202 OA risk genes, we identified 29 suggestively significant KEGG pathways (unadjusted *P* < 0.05). Here, we provide the top 20 significant pathways, as provided in **Table 1**. The transforming growth factor β (TGF-β) signaling pathway is the most significant signal (*P* = 5.98E−05). Importantly, this is the only pathway after the BH multiple-test adjustment with FDR = 0.02. In addition, we identified other OA risk pathways including inflammatory bowel disease (IBD), human T-cell leukemia virus 1 infection, RA, influenza A, asthma, tuberculosis, prostate cancer, hematopoietic cell lineage, transcriptional misregulation in cancer, allograft rejection, mitogen-activated protein kinase (MAPK) signaling pathway, T_H_7 cell differentiation, graft-versus-host disease, toxoplasmosis, type 1 diabetes mellitus, cell cycle, intestinal immune network for IgA production, autoimmune thyroid disease, and ubiquitin-mediated proteolysis. More detailed information is described in **Table 1**.

**Table 1:** Top 20 osteoarthritis risk pathways identified in KEGG database.

Gene set	Description	Size	Expect	Ratio	*P*	FDR
hsa04350	TGF-β signaling pathway	84	0.43	11.70	5.98E−05	0.02
hsa05321	Inflammatory bowel disease	65	0.33	9.07	4.27E−03	0.41
hsa05166	Human T-cell leukemia virus 1 infection	255	1.30	3.85	8.91E−03	0.41
hsa05323	Rheumatoid arthritis	90	0.46	6.55	1.05E−02	0.41
hsa05164	Influenza A	171	0.87	4.60	1.07E−02	0.41
hsa05310	Asthma	31	0.16	12.68	1.07E−02	0.41
hsa05152	Tuberculosis	179	0.91	4.39	1.25E−02	0.41
hsa05215	Prostate cancer	97	0.49	6.08	1.29E−02	0.41
hsa04640	Hematopoietic cell lineage	97	0.49	6.08	1.29E−02	0.41
hsa05202	Transcriptional misregulation in cancer	186	0.95	4.23	1.42E−02	0.41
hsa05330	Allograft rejection	38	0.19	10.35	1.58E−02	0.41
hsa04010	Mitogen-activated protein kinase signaling pathway	295	1.50	3.33	1.60E−02	0.41
hsa04659	T_H_17 cell differentiation	107	0.54	5.51	1.68E−02	0.41
hsa05332	Graft-versus-host disease	41	0.21	9.59	1.82E−02	0.41
hsa05145	Toxoplasmosis	113	0.57	5.22	1.94E−02	0.41
hsa04940	Type 1 diabetes mellitus	43	0.22	9.14	2.00E−02	0.41
hsa04110	Cell cycle	124	0.63	4.76	2.47E−02	0.46
hsa04672	Intestinal immune network for IgA production	49	0.25	8.02	2.55E−02	0.46
hsa05320	Autoimmune thyroid disease	53	0.27	7.42	2.95E−02	0.51
hsa04120	Ubiquitin-mediated proteolysis	137	0.70	4.30	3.19E−02	0.51

### Identification of OA-Related GO Biological Processes

Using these 202 OA risk genes, we identified 24 statistically significant GO biological processes (FDR < 0.05). These biological processes could be mainly divided into two classes including differentiation and development. The differentiation-related biological processes consist of chondrocyte differentiation, regulation of chondrocyte differentiation, positive regulation of chondrocyte differentiation, regulation of epithelial cell proliferation, regulation of cell proliferation, and epithelial cell proliferation. The development-related biological processes consist of regulation of cartilage development, cartilage development, connective tissue development, chondrocyte development, skeletal system development, liver development, hepaticobiliary system development, positive regulation of cartilage development, striated muscle tissue development, and muscle tissue development. Here, we provide the top 20 significant biological processes, as provided in **Table 2**.

**Table 2:** Top 20 osteoarthritis risk biological processes identified in GO database.

Gene set	Description	Size	Expect	Ratio	*P*	FDR
GO:0002062	Chondrocyte differentiation	119	0.7497	12.004	6.01E−08	5.00E−04
GO:0061035	Regulation of cartilage development	65	0.4095	17.093	1.66E−07	8.00E−04
GO:0032330	Regulation of chondrocyte differentiation	47	0.2961	20.263	4.71E−07	1.40E−03
GO:0051216	Cartilage development	205	1.2916	7.7426	6.65E−07	1.50E−03
GO:0061448	Connective tissue development	265	1.6696	6.5885	8.89E−07	1.60E−03
GO:0032332	Positive regulation of chondrocyte differentiation	20	0.126	31.745	6.67E−06	1.01E−02
GO:0002063	Chondrocyte development	46	0.2898	17.253	0	1.31E−02
GO:0001501	Skeletal system development	506	3.1879	4.0779	0	1.98E−02
GO:0001503	Ossification	371	2.3374	4.7061	0	2.21E−02
GO:0001889	Liver development	137	0.8631	8.11	0	2.21E−02
GO:0070848	Response to growth factor	690	4.3472	3.4505	0	2.21E−02
GO:0061008	Hepaticobiliary system development	140	0.882	7.9362	0	2.21E−02
GO:0050678	Regulation of epithelial cell proliferation	318	2.0035	4.9913	0	2.25E−02
GO:0009887	Animal organ morphogenesis	974	6.1364	2.9333	0	2.25E−02
GO:0061036	Positive regulation of cartilage development	31	0.1953	20.48	0	2.40E−02
GO:0090100	Positive regulation of transmembrane receptor protein serine/threonine kinase signaling pathway	101	0.6363	9.4291	0	2.40E−02
GO:0060389	Pathway-restricted SMAD protein phosphorylation	64	0.4032	12.4	1.00E−04	2.74E−02
GO:0014706	Striated muscle tissue development	357	2.2492	4.446	1.00E−04	4.10E−02
GO:0090092	Regulation of transmembrane receptor protein serine/threonine kinase signaling pathway	224	1.4113	5.6687	1.00E−04	4.10E−02
GO:0042127	Regulation of cell proliferation	1564	9.8536	2.3342	1.00E−04	4.13E−02

### Identification of OA-Related GO Molecular Functions

Using these 202 OA risk genes, we identified only one statistically significant GO molecular function (FDR < 0.05) TGF-β receptor binding (*P* = 9.30E−06). Here, we provide the top 20 significant molecular functions, as provided in **Table 3**. Most of these molecular functions are related with binding including TGF-β receptor binding, phospholipid binding, TGF-β binding, peptide antigen binding, antigen binding, phosphatase binding, type II TGF-β receptor binding, protein phosphatase binding, lipid binding, bridging protein binding, apolipoprotein binding, cytokine receptor binding, and cytokine binding.

**Table 3:** Top 20 osteoarthritis risk molecular functions identified in GO database.

Gene set	Description	Size	Expect	Ratio	*P*	FDR
GO:0005160	Transforming growth factor β receptor binding	51	0.28	17.57	9.30E−06	0.02
GO:0005543	Phospholipid binding	412	2.30	4.35	1.00E−04	0.09
GO:0050431	Transforming growth factor β binding	22	0.12	24.44	2.00E−04	0.09
GO:0042605	Peptide antigen binding	22	0.12	24.44	2.00E−04	0.09
GO:0003823	Antigen binding	55	0.31	13.04	2.00E−04	0.09
GO:0008083	Growth factor activity	163	0.91	6.60	3.00E−04	0.09
GO:0019902	Phosphatase binding	178	0.99	6.04	5.00E−04	0.13
GO:0005114	Type II transforming growth factor β receptor binding	7	0.04	51.22	6.00E−04	0.15
GO:0019903	Protein phosphatase binding	133	0.74	6.74	9.00E−04	0.19
GO:0003777	Microtubule motor activity	83	0.46	8.64	1.20E−03	0.22
GO:0005201	Extracellular matrix structural constituent	158	0.88	5.67	1.90E−03	0.33
GO:0008289	Lipid binding	718	4.01	2.75	2.10E−03	0.34
GO:0030674	Protein binding, bridging	172	0.96	5.21	2.80E−03	0.39
GO:0034185	Apolipoprotein binding	15	0.08	23.90	3.10E−03	0.39
GO:0005024	Transforming growth factor β–activated receptor activity	15	0.08	23.90	3.10E−03	0.39
GO:0001228	DNA-binding transcription activator activity, RNA polymerase II specific	444	2.48	3.23	3.40E−03	0.39
GO:0004675	Transmembrane receptor protein serine/threonine kinase activity	17	0.09	21.09	4.00E−03	0.44
GO:0005126	Cytokine receptor binding	274	1.53	3.93	4.30E−03	0.45
GO:0060090	Molecular adaptor activity	194	1.08	4.62	4.60E−03	0.46
GO:0019955	Cytokine binding	127	0.71	5.65	5.60E−03	0.52

### Identification of OA-Related GO Cellular Components

Using these 202 OA risk genes, we identified five statistically significant GO cellular components (FDR < 0.05) including extracellular matrix, COPII-coated endoplasmic reticulum (ER) to Golgi transport vesicle, collagen-containing extracellular matrix, extracellular matrix component, and major histocompatibility complex (MHC) protein. In addition, the top 20 significant cellular components also include coated vesicle, nucleoplasm part, endocytic vesicle membrane, fibrillar collagen trimer, banded collagen fibril, Golgi-associated vesicle, MHC class II protein complex, ER to Golgi transport vesicle membrane, cytoplasmic vesicle membrane, ER lumen, vesicle membrane, complex of collagen trimers, ER part, microtubule associated complex, and sarcoplasm. More detailed information is described in **Table 4**.

**Table 4:** Top 20 osteoarthritis risk cellular components identified in GO database.

Gene set	Description	Size	Expect	Ratio	*P*	FDR
GO:0031012	Extracellular matrix	496	2.37	4.65	0	0.02
GO:0030134	COPII-coated endoplasmic reticulum (ER) to Golgi transport vesicle	87	0.42	12.04	1.00E−04	0.02
GO:0062023	Collagen-containing extracellular matrix	366	1.75	5.15	1.00E−04	0.02
GO:0044420	Extracellular matrix component	49	0.23	17.10	1.00E−04	0.03
GO:0042611	Major histocompatibility complex (MHC) protein complex	21	0.10	29.93	1.00E−04	0.03
GO:0030135	Coated vesicle	275	1.31	5.33	3.00E−04	0.07
GO:0044451	Nucleoplasm part	1087	5.19	2.70	6.00E−04	0.10
GO:0030666	Endocytic vesicle membrane	160	0.76	6.55	1.00E−03	0.14
GO:0005583	Fibrillar collagen trimer	11	0.05	38.09	1.20E−03	0.14
GO:0098643	Banded collagen fibril	11	0.05	38.09	1.20E−03	0.14
GO:0005798	Golgi-associated vesicle	169	0.81	6.20	1.30E−03	0.14
GO:0042613	MHC class II protein complex	15	0.07	27.93	2.30E−03	0.22
GO:0012507	ER to Golgi transport vesicle membrane	57	0.27	11.03	2.60E−03	0.23
GO:0030659	Cytoplasmic vesicle membrane	746	3.56	2.81	2.90E−03	0.24
GO:0005788	Endoplasmic reticulum lumen	306	1.46	4.11	3.40E−03	0.25
GO:0012506	Vesicle membrane	767	3.66	2.73	3.50E−03	0.25
GO:0098644	Complex of collagen trimers	19	0.09	22.05	3.70E−03	0.25
GO:0044432	Endoplasmic reticulum part	1332	6.36	2.20	4.00E−03	0.26
GO:0005875	Microtubule associated complex	148	0.71	5.66	5.50E−03	0.34
GO:0016528	Sarcoplasm	77	0.37	8.16	6.00E−03	0.34

### OA Case–Control Gene Expression Analysis

Of the selected 202 risk genes, 122 risk genes were included in the OA case–control gene expression dataset, as provided in **Supplementary Table 4**. The results showed that 44 of these 122 risk genes showed suggestively differential expression in OA cases compared with controls (*P* < 0.05). Importantly, three genes including WWP2, COG5, and MAPT showed statistically different expression in OA cases compared with controls (*P* < 0.05/122 = 4.10E−04), as described in **Table 5**.

**Table 5:** Forty-four significantly differentially expressed osteoarthritis risk genes.

ID	*T*	*B*	Fold change	Gene	*P*
ILMN_1659703	−4.6029458	3.22309	0.87	WWP2	9.31E−06
ILMN_1721535	4.0329713	1.10042	1.26	COG5	9.05E−05
ILMN_1800049	−3.68066	−0.10141	0.96	MAPT	3.32E−04
ILMN_1804895	−3.4402192	−0.86996	0.92	LSMEM1	7.68E−04
ILMN_1778886	−3.397218	−1.00284	0.92	ZNF345	8.89E−04
ILMN_1738239	−3.3661954	−1.09784	0.84	RBM6	9.86E−04
ILMN_1698621	3.1752911	−1.66604	1.07	COG5	1.84E−03
ILMN_1768261	−3.0820359	−1.93322	0.95	PRDM2	2.48E−03
ILMN_1673708	2.9367424	−2.33566	1.02	HDAC9	3.88E−03
ILMN_1776858	−2.924587	−2.36856	0.93	DUS4L	4.03E−03
ILMN_1772218	2.8667893	−2.52332	1.12	HLA-DPA1	4.79E−03
ILMN_1785402	−2.7406448	−2.85157	0.95	LTBP1	6.94E−03
ILMN_1790384	−2.7296251	−2.87962	0.96	PRDM2	7.16E−03
ILMN_1802973	−2.7280423	−2.88364	0.87	ANAPC4	7.20E−03
ILMN_1699469	2.6997749	−2.95509	1.03	KCNIP4	7.80E−03
ILMN_1749026	−2.6961724	−2.96415	0.98	LCT	7.88E−03
ILMN_1693559	2.678254	−3.00904	1.02	DOT1L	8.29E−03
ILMN_1690442	−2.6764894	−3.01345	0.97	TMEM241	8.34E−03
ILMN_2381121	2.6586005	−3.05797	1.06	UQCC1	8.77E−03
ILMN_1682981	−2.6104688	−3.17642	0.95	SMG6	1.00E−02
ILMN_1753353	2.5464314	−3.33099	1.07	SLBP	1.20E−02
ILMN_1823056	−2.4053844	−3.65907	0.98	CCDC33	1.75E−02
ILMN_1716651	2.4049149	−3.66013	1.07	RUNX2	1.75E−02
ILMN_2408885	2.3961067	−3.68005	1.03	HDAC9	1.79E−02
ILMN_1701361	−2.3821458	−3.71148	0.98	LURAP1L	1.86E−02
ILMN_1754121	2.3523213	−3.77806	1.09	CSK	2.01E−02
ILMN_1693427	−2.3253143	−3.83768	0.98	GLIS3	2.15E−02
ILMN_1747386	−2.3074266	−3.87682	0.98	GLIS3	2.25E−02
ILMN_1717780	2.2917001	−3.911	1.02	PLEC	2.34E−02
ILMN_1771987	2.2874158	−3.92028	1.12	SLC44A2	2.37E−02
ILMN_2145670	2.2761251	−3.94464	1.03	TNC	2.44E−02
ILMN_1784287	2.2239902	−4.05569	1.17	TGFBR3	2.78E−02
ILMN_2129668	−2.200499	−4.10495	0.98	TGFB1	2.94E−02
ILMN_1780291	−2.1782335	−4.15119	0.95	NFAT5	3.11E−02
ILMN_1882354	2.1649163	−4.17863	1.15	FAM53A	3.21E−02
ILMN_1680399	−2.1603694	−4.18797	0.97	KAZN	3.25E−02
ILMN_1726387	−2.1502554	−4.20866	0.97	NF1	3.33E−02
ILMN_1654421	−2.129798	−4.25025	0.94	MPHOSPH9	3.50E−02
ILMN_1724734	2.1181634	−4.27374	1.03	UQCC1	3.59E−02
ILMN_2381559	2.0754774	−4.35887	1.02	ASTN2	3.98E−02
ILMN_1673620	−2.0711976	−4.36732	0.97	KIF26B	4.02E−02
ILMN_1871893	−2.0480746	−4.41267	0.98	LINC01507	4.24E−02
ILMN_2046073	2.0412532	−4.42596	1.02	LCT	4.31E−02
ILMN_1813277	2.0065873	−4.49284	1.11	SUPT3H	4.67E−02

T, Moderated t-statistic; B, B-statistic or log-odds that the gene is differentially expressed; F, Moderated F-statistic combines the t-statistics for all the pair-wise comparisons into an overall test of significance for that gene.

## Discussion

In this study, we first selected 187 independent genetic variants identified by GWAS (*P* < 1.00E−05) and found that most of these genetic variants are noncoding mutations. We then conducted an eQTLs analysis and found that 165 of these 187 genetic variants could significantly regulate the expression of nearby genes. Third, we identified OA susceptibility genes corresponding to these genetic variants, conducted a pathway analysis, and identified novel OA-related KEGG pathways, GO biological processes, GO molecular functions, and GO cellular components.

In KEGG database, TGF-β signaling pathway is the most significant signal (*P* = 5.98E−05) and is the only pathway after the BH multiple-test adjustment with FDR = 0.02. Our finding is consistent with previous findings. Recent findings provide substantial evidence that TGF-β signaling contributes to OA development and progression (Shen et al., 2014; Fang et al., 2016). In addition, we found the association of OA with other human diseases including IBD, RA, asthma, prostate cancer, hematopoietic cell lineage, transcriptional misregulation in cancer, allograft rejection, MAPK signaling pathway, graft-versus-host disease, type 1 diabetes mellitus, and autoimmune thyroid disease. Previous study supported our finding about the association of human T-cell leukemia virus 1 infection pathway with OA. It is reported that human T lymphotropic virus type I retrovirus infection could alter the expression of inflammatory cytokines in primary OA (Yoshihara et al., 2004). Here, we highlighted the association of T_H_17 cell differentiation pathway with OA. Evidence showed that complement could drive T_H_17 cell differentiation and trigger autoimmune arthritis (Hashimoto et al., 2010; Li et al., 2017).

In GO database, we identified 24 statistically significant GO biological processes, one statistically significant GO molecular function, and five statistically significant GO cellular components (FDR < 0.05). These signals are related with chondrocyte differentiation and development, which are all known biological pathways associated with OA. Take the chondrocyte differentiation (GO:0002062), for example, previous studies also supported the hypertrophic differentiation of chondrocytes in OA (Dreier, 2010; Goldring, 2012). Some biological pathways are related with TGF-β signaling binding, such as TGF-β receptor binding, TGF-β binding, and type II TGF-β receptor binding. The phospholipid binding (GO:0005543) is the second significant signal among the top 20 OA risk molecular functions identified in GO database. Evidence shows that lipids are important nutrients in chondrocyte metabolism (Villalvilla et al., 2013). The lipid availability is important to keep cartilage status and OA development (Villalvilla et al., 2013).

Using an OA case–control gene expression analysis, we showed that 44 of these 122 risk genes were suggestively differentially expressed in OA cases compared with controls (*P* < 0.05). Three genes, WWP2, COG5, and MAPT, were statistically differentially expressed in OA cases compared with controls (*P* < 0.05/122 = 4.10E−04).

In summary, we believe that these findings provide further insight into the underlying genetic mechanisms for these newly identified OA risk genes. Although these are interesting findings, we also realize some limitations to our study. Until now, pathway analyses of RA GWAS datasets have widely been reported, but not OA GWAS datasets (Eleftherohorinou et al., 2009; Eleftherohorinou et al., 2011; Lee et al., 2012b; Song et al., 2013; Zhang et al., 2016). In the future, we will compare our findings using top OA genetic variants with pathway analysis of OA using the whole GWAS datasets and further clarify the differences between top OA genetic variants and the whole OA GWAS datasets. In addition, a gene expression analysis in OA-related tissues is necessary to demonstrate that these pathways are deregulated in OA cases and controls. However, this kind of gene expression datasets in OA-related tissues is not available now. Hence, we will further evaluate our findings using gene expression datasets in OA-related tissues in the future.

## Data Availability

Publicly available datasets were analyzed in this study. This data can be found here: https://www.ebi.ac.uk/gwas/downloads/summary-statistics.

## Author Contributions

FG and JT conceived and initiated the project. FG analyzed the data. All authors wrote and reviewed the manuscript.

## Funding

This work was supported by funding from Heilongjiang Natural Science Foundation (grant no. H2016025).

## Conflict of Interest Statement

The authors declare that the research was conducted in the absence of any commercial or financial relationships that could be construed as a potential conflict of interest.
